# Editorial: Electrical signals in leaves - mirrors of health and physiological activities in plants

**DOI:** 10.3389/fpls.2024.1458116

**Published:** 2024-07-18

**Authors:** Vladimir Sukhov, Yanyou Wu, Deke Xing, Lan Huang

**Affiliations:** ^1^ Department of Biophysics, N.I. Lobachevsky State University of Nizhny Novgorod, Nizhny Novgorod, Russia; ^2^ State Key Laboratory of Environmental Geochemistry, Institute of Geochemistry, Chinese Academy of Sciences, Guiyang, China; ^3^ Institute of Agricultural Engineering, Jiangsu University, Zhenjiang, China; ^4^ College of Information and Electrical Engineering, China Agricultural University, Beijing, China

**Keywords:** electrophysiological parameters, electrical stress physiology, water transport, nutrient use efficiency, photosynthesis, growth

The plant cell membrane has strict selective permeability to various ions (Yu et al., 2021). The electrolyte solution on both sides of the membrane forms a specific conductive state, which is equivalent to the bipolar plate of the capacitor, and the cell membrane is equivalent to the intermediate medium of the capacitor, showing capacitance. The organelles and the cytoplasm in a cell are regarded as resistors. Electric current is influenced by the resistors, capacitors, and inductors in the alternating current circuit, and impedance is the sum of the resistance to current caused by the resistors, capacitors, and inductors (Schönleber and Ivers-Tiffée, 2015). In the process of cell damage, cell structure, composition, and ion permeability will undergo complex changes, resulting in significant changes in electrical characteristics (Nguyen et al., 2018). As a newly emerging sensor technology, electrophysiology is sensitive to environmental changes and has been increasingly used for monitoring plant responses to the environment (Steeneken et al., 2023).

Under natural conditions, terrestrial plants can be affected by adverse abiotic and biotic factors which induce stress signals and trigger adaptive responses. Electrical signals, which are dependent on many plant processes and induce numerous physiological changes, can participate in the fast adaptive responses (Gallé et al., 2015; Hedrich et al., 2016). Therefore, investigations into the electrical activity in terrestrial plants can potentially provide new tools for managing plant productivity under adverse conditions and for estimating the health of plants through the analysis of their electrical parameters. These inquiries should include an analysis of molecular mechanisms of the plant electrical activity (particularly, participation of ion channels and active ion transporters), a study of relations between the electrical activity and specific stressors, the effects of electrical signals on specific physiological processes, method development for the analysis of electrical records, and generation of mathematical models of the electrical activity, among other things. Thus, the aim of this Research Topic was to highlight these points.

A study by Han et al. was devoted to the analysis of the participation of K^+^ channel ZMK1 (*Zea mays* K^+^ channel 1), a maize ortholog of the Arabidopsis AKT1 channel (Arabidopsis K^+^ Transporter1), in K^+^ uptake by roots and potassium homeostasis in the plant. By using heterologous systems (Xenopus oocytes and yeast) and transgenic lines of Arabidopsis and maize, it was shown that ZMK1, together with the protein kinase ZmCIPK23 (Zea mays calcineurin B-like protein-interacting serine/threonine-protein kinase 23) which activates the K^+^ channel, plays an important role in these processes.

Teng et al. identified two jasmonic acid carboxyl methyltransferases, GhJMT1 and GhJMT2, which participate in methyl jasmonate biosynthesis from the jasmonic acid in *Gossypium hirsutum*, and analyzed their functional activity. It was shown that *GhJMT1* and *GhJMT2* are up-regulated by the methyl jasmonate treatment. The treatment also induces emission of (E)-β-ocimene, (Z)-3-hexenyl acetate, linalool, and (3E)-4,8-dimethyl-1,3,7-nonatriene (DMNT), which participate in plant defense. Specifically, these volatiles and the methyl jasmonate induce electrophysiological responses in the antennae of *Microplitis*, parasitoid wasps. The results clarify ways of regulating the concentrations of methyl jasmonate and jasmonic acid, suggesting ways these can be employed in agricultural pest control. Considering the positive influence of electrical signals on jasmonic acid concentration (Hlavácková et al., 2006), the results could also be used for the analysis of the effects of these signals on plant defenses.

Parise et al. showed that the content of photosynthetic pigments in the parasitic plant dodder (*Cuscuta racemosa* Mart.) is dependent on the absence or presence of host species. The effect is likely to be related to the complex plant electrical activity (electrome) because these species can be detected on the basis of an electrophysiological time series recording analyzed by machine learning techniques. These results suggest that electrical signaling can participate in parasitic plant foraging and may be involved in the attraction of dodder parasites to the host.

Yudina et al. investigated the effects of hyperpolarization electrical signals induced by moderate heating (to 40°C) and illumination on wheat plant photosynthesis. It was shown that these signals decrease the quantum yield of photosystem II and stimulate the non-photochemical quenching of chlorophyll fluorescence. Moderate drought stimulates the photosynthetic response and increases the amplitude of electrical signals. In contrast, strong drought suppresses both the photosynthetic response and the electrical signals. Thereby, hyperpolarization electrical signals, which are induced by widespread stressors, can affect photosynthesis in terrestrial plants.

Liu et al. investigated the effect of root cutting on the characteristics of photosynthetic light reactions in leaves of *Artemisia ordosica* Krasch measured via an OJIP-test, a method of analysis of the chlorophyll fluorescence induction curve, two days after cutting. It was shown that moderate root cutting (less than 30%) weakly affects photosynthetic light reactions. In contrast, more severe root cutting (greater than 30%) prominently influences the OJIP test parameters, showing a reduced energy capture and electron transfer efficiency in photosystem II. The results suggest that the OJIP test parameters can be used to reveal the severity of root cutting. Additionally, they indicate that stress signals propagated from roots to leaves affect photosynthesis; these signals can be electrical signals (variation potentials) because mechanical damage (e.g., the cutting) is one of the mechanisms through which variation potential is induced (Li et al., 2021).

Finally, a study by Xing et al. was devoted to the analysis of the role of water retained inside the leaf in the regulation of photosynthesis and growth in tomatoes under water deficit. The authors used an original model on the basis of the Nernst equation to estimate water content from passive electrical properties of the leaf (a physiological impedance). The results of this work indicate an increased transport rate of leaf intracellular water under water deficit. The enhanced transport rate is likely to help plants use intracellular water more efficiently, enabling plant growth and photosynthesis under drought conditions.

Thus, the studies published in the Research Topic, “Electrical Signals in Leaves - Mirrors of Health and Physiological Activities in Plants”, clarify different aspects of the electrical signaling in plants: from ion mechanisms of potassium transport (K^+^ is an important ion in the electrical activity) to electrophysiological activity of the whole plant. The cross-disciplinary approaches combined with electrophysiology and molecular biology will lead to a deeper understanding of plant physiological processes.

## Author contributions

VS: Writing – original draft. YW: Writing – review & editing. DX: Writing – review & editing. LH: Writing – review & editing.
